# Radiosynthesis and preclinical evaluation of [^11^C]prucalopride as a potential agonist PET ligand for the 5-HT_4_ receptor

**DOI:** 10.1186/2191-219X-3-24

**Published:** 2013-04-04

**Authors:** Hans JC Buiter, Albert D Windhorst, Marc C Huisman, Joris H De Maeyer, Jan AJ Schuurkes, Adriaan A Lammertsma, Josée E Leysen

**Affiliations:** 1 Department of Radiology and Nuclear Medicine, VU University Medical Center, PO Box 7057, Amsterdam, MB, 1007, The Netherlands; 2 Shire-Movetis NV, Veedijk 58 (1004), Turnhout, 2300, Belgium

**Keywords:** [^11^C]Prucalopride, Radiosynthesis, 5-HT_4_ receptor, Agonist, G-protein coupled receptor, Rat, Biodistribution, PET imaging

## Abstract

**Background:**

Serotonin 5-HT_4_ receptor (5-HT_4_-R) agonists are potential therapeutic agents for enterokinetic and cognitive disorders and are marketed for treatment of constipation. The aim of this study was to develop an agonist positron emission tomography (PET) ligand in order to label the active G-protein coupled 5-HT_4_-R in peripheral and central tissues. For this purpose prucalopride, a high-affinity selective 5-HT_4_-R agonist, was selected.

**Methods:**

[^11^C]Prucalopride was synthesized from [^11^C]methyl triflate and desmethyl prucalopride, and its LogD_oct,pH7.4_ was determined. Three distinct studies were performed with administration of IV [^11^C]prucalopride in male rats: (1) The biodistribution of radioactivity was measured *ex vivo*; (2) the kinetics of radioactivity levels in brain regions and peripheral organs was assessed *in vivo* under baseline conditions and following pre-treatment with tariquidar, a P-glycoprotein efflux pump inhibitor; and (3) *in vivo* stability of [^11^C]prucalopride was checked *ex vivo* in plasma and brain extracts using high-performance liquid chromatography.

**Results:**

[^11^C]Prucalopride was synthesized in optimised conditions with a yield of 21% ± 4% (decay corrected) and a radiochemical purity (>99%), its LogD_oct,pH7.4_ was 0.87. *Ex vivo* biodistribution studies with [^11^C]prucalopride in rats showed very low levels of radioactivity in brain (maximal 0.13% ID·g^−1^) and ten times higher levels in certain peripheral tissues. The PET studies confirmed very low brain levels of radioactivity under baseline conditions; however, it was increased three times after pre-treatment with tariquidar. [^11^C]Prucalopride was found to be very rapidly metabolised in rats, with no parent compound detectable in plasma and brain extracts at 5 and 30 min following IV administration. Analysis of levels of radioactivity in peripheral tissues revealed a distinct PET signal in the caecum, which was reduced following tariquidar pre-treatment. The latter is in line with the role of the P-glycoprotein pump in the gut.

**Conclusion:**

[^11^C]Prucalopride demonstrated low radioactivity levels in rat brain; a combination of reasons may include rapid metabolism in the rat in particular, low passive diffusion and potential P-glycoprotein substrate. In humans, further investigation of [^11^C]prucalopride for imaging the active state of 5-HT_4_-R is worthwhile, in view of the therapeutic applications of 5-HT_4_ agonists for treatment of gastrointestinal motility disorders.

## Background

In mammalian species, serotonin or 5-hydroxytryptamine (5-HT) acts as a neurotransmitter and paracrine agent that mediates a wide variety of functions, including cognitive and emotional processes, regulation of sleep and food intake and cardiovascular and gastrointestinal mechanisms. Serotonergic neurons originate in the raphé nuclei of the brain stem and project widely to forebrain, hind brain and spinal cord. The 5-HT is also synthesized in enterochromaffin cells in the gut. The latter contains 90% of the 5-HT in the body, from where it is released in the blood to exert a paracrine actions. To date 14 different 5-HT receptors, classified into seven subclasses, have been identified. The 5-HT_1A/1B/1D/1E/1F_, 5-HT_2A/2B/2C_, 5-HT_4_, 5-ht_5A/5B_, 5-HT_6_ and 5-HT_7_ receptors are G-protein coupled receptors (GPCR); the 5-HT_3_ receptor belongs to the class of ligand-gated ion channels [1].

The 5-HT_4_ receptor (5-HT_4_-R) is of interest for its role in the central nervous system (CNS) and in the peripheral tissues. For the latter, 5-HT_4_-R agonists are applied therapeutically to treat laxative-resistant constipation [2], and in the CNS, 5-HT_4_-R agonists have been shown to improve memory and cognition in animal models [3,4]. In the human brain, 5-HT_4_-Rs have been localized in the basal ganglia, the hippocampal formation and the cortical mantle [5].

A non-invasive molecular imaging technique such as positron emission tomography (PET) could be useful to explore the function of 5-HT_4_-R *in vivo*. To date only [^11^C]SB207145, a high-affinity 5-HT_4_-R antagonist [6,7], has been evaluated in large mammals and man. In general, successful PET ligands for GPCRs are antagonists; nevertheless, antagonists have an important disadvantage. GPCRs are known to occur in interconvertible active G-protein coupled states and in inactive uncoupled or desensitised states. Antagonists show equal affinity for these different GPCR states and consequently do not distinguish between the active and the inactive receptor. In contrast, agonists have high affinity for the active G-protein coupled receptors and low affinity for inactive uncoupled receptors. Therefore, agonist PET ligands will preferentially label the activated receptors [8].

Prucalopride is a potent selective 5-HT_4_-R agonist [9], currently marketed for human use for the treatment of laxative-resistant constipation [2]. Studies with [^3^H]prucalopride demonstrated favourable radioligand binding properties *in vitro*[5]. Affinity was high (*K*_D_ = 2 nM) and using autoradiography *in vitro* [^3^H]prucalopride clearly labelled 5-HT_4_-R in striatum, hippocampus, frontal cortex and substantia nigra in human brain hemispheres cryosections [5]. Comparison of 5-HT_4_-R densities measured with the agonist [^3^H]prucalopride and with the antagonist [^3^H]R116712 in various brain regions revealed that *B* max values measured with the former, represented 16% to 54% of the *B* max values of the latter [5]. This indicates that the active G-protein coupled 5-HT_4_-R state may vary between various brain regions and potentially also between peripheral tissues. This illustrates the importance of developing an agonist 5-HT_4_-R PET ligand that would allow investigating the active 5-HT_4_-R state *in vivo*.

The aim of the present study was to label prucalopride with carbon-11, optimize its radiosynthesis and investigate [^11^C]prucalopride in biodistribution *ex vivo* and *in vivo* studies in rats.

## Methods

### Chemicals

Prucalopride succinate (4-amino-5-chloro-*N*-[1-(3-methoxypropyl)piperidin-4-yl]-2,3-dihydro-1-benzofuran-7-carboxamide monobutanedioate) and desmethyl prucalopride (4-amino-5-chloro-*N*-[1-(3-hydroxypropyl)piperidin-4-yl]-2,3-dihydro-1-benzofuran-7-carboxamide) were provided by Shire-Movetis NV (Turnhout, Belgium). Tariquidar, a P-glycoprotein drug efflux pump inhibitor [10-13], was obtained from Haupt Pharma Wülfing GmbH (Gronau, Germany). All other reagents were from Merck (Schiphol-Rijk, The Netherlands) or Sigma-Aldrich (Zwijndrecht, The Netherlands). They were of analytical grade and used without further purification. High-performance liquid chromatography (HPLC) solvents (gradient grade) were purchased from Mallinckrodt-Baker BV (Deventer, The Netherlands).

### High-performance liquid chromatography

The semi-preparative HPLC system consisted of a Jasco (Ishikawa-cho, Japan) PU-1587 HPLC pump, a six-way VICI injector (VA EPC6W, VICI AG International, Schenkon, Switzerland) with a 5-mL loop, a Jasco UV1575 UV detector, a custom made radioactivity detector and a Phenomenex (Torrance, CA, USA) Synergi 10 μm hydro-RP 80 C18 250 × 10 mm HPLC column; and an eluent of methanol/0.1 M phosphate buffer (pH 4.3) 34/66 (*v/v*) was used with a flow of 6 mL·min^−1^. Radioactivity was measured using a Veenstra (Joure, The Netherlands) VDC-405 dose calibrator. The analytical HPLC system consisted of a Jasco PU-1580 HPLC pump, a Rheodyne 7724I injector (IDEX Health & Science, Wertheim-Mondfeld, Germany) with a 20-μL loop, a Jasco UV-2075 Plus UV detector, a NaI radioactivity detector (Raytest, Straubenhardt, Germany) and a Phenomenex Gemini 5 μm C18 150 × 4.6 mm column; and an eluent of methanol/0.1 M phosphate buffer (pH 4.3) 30/70 (*v/v*) was used with at a flow of 1 mL min^−1^. The peak of parent prucalopride was identified by co-injection of a sample of cold prucalopride.

### Preparation of [^11^C]prucalopride

The automated radiolabelling [14] of [^11^C]prucalopride was performed by addition of [^11^C]methyltriflate [15] to a solution of 350 μL acetonitrile containing 1.0 mg (2.7 μmol) desmethyl prucalopride and 1.9 μL (4.3 μmol) tetrabutylammoniumhydroxide (60% solution in water) at −25°C. After distillation and trapping of [^11^C]methyltriflate, the temperature was raised to 85°C for 5 min, after which the reaction mixture was quenched with 0.4 mL of phosphate buffer (0.1 M, pH 4.3) and diluted with 1.5 mL of HPLC eluent. This mixture was subjected to semi-preparative HPLC purification. The fraction containing [^11^C]prucalopride was collected and diluted with 60 mL of sterile water.

The product was trapped by solid phase extraction using a pre-conditioned (10 mL of ethanol followed by 10 mL of sterile water) Waters tC18 Plus Sep Pak® cartridge (Waters Chromatography BV, Etten-Leur, The Netherlands), which was subsequently washed with 20 mL sterile water. Next, the product was eluted from the Sep Pak cartridge with 1 mL ethanol followed by 9 mL saline containing 7.09 mM NaH_2_PO_4_ and filtered through a sterile Millex GV 0.22 μm membrane filter (Millipore BV, Amsterdam, the Netherlands), using helium overpressure. Devices used for the automated radiosyntheses were homemade [14]. The purity of the product was analysed using the analytical HPLC system, and specific activity (SA) was determined by HPLC based on a calibration curve (performed in triplicate with four concentrations of parent prucalopride in the range of 1.10^−4^ to 1.10^−7^ mol L^−1^) and measurement of the ultraviolet (UV) signal.

### Determination of the LogD_oct,pH7.4_ value of [^11^C]prucalopride

The distribution of [^11^C]prucalopride between 1-octanol and 0.2 M phosphate buffer (pH 7.4) was measured in triplicate at room temperature by adapting a method previously described [16]. Briefly, 1 mL of 10 MBq·mL^−1^ solution of [^11^C]prucalopride in 0.2 M phosphate buffer (pH = 7.4) was mixed vigorously with 1 mL 1-octanol for 1 min at room temperature using a vortex. After centrifugation for 5 min at 4,000 rpm and a settling period of 30 min, five aliquots of 100 μL were taken from both layers, carefully avoiding cross contamination between the phases. Five aliquots of 100 μL of the 10 MBq·mL^−1^ solution of [^11^C]prucalopride in 0.2 M phosphate buffer (pH = 7.4) were taken as reference for determining recovery. All aliquots were counted for radioactivity using an automated gamma counter, Wallac 1282 Compugamma CS (LKB Wallac, Turku, Finland). The LogD_oct,pH7.4_ value was calculated according to LogD_oct,pH7.4_ = ^10^Log(*A*_oct_ / *A*_buffer_), with *A*_buffer_ as the average radioactivity of five buffer samples and *A*_oct_ as the average radioactivity of 5 1-octanol samples.

### Drug solutions

[^11^C]prucalopride and [^18^F]NaF, prepared as described previously [17], were diluted with saline to prepare an isotonic, sterile and pyrogen-free solution for IV injection. Radiochemical purity was >99%.

A tariquidar solution in 20% ethanol was diluted with 5% glucose in saline to a concentration of 7.5 mg/mL for IV injection. The final concentration of ethanol was 10% for the PET studies.

### Animals

Experiments were performed using male Wistar rats (265 ± 15 g; Harlan Netherlands BV Horst, The Netherlands). Rats were kept in conditioned housing under a regular light/dark cycle (12/12 h) and allowed food and water *ad libitum*. All animal experiments were in compliance with Dutch law and approved by the VU University Animal Ethics Committee.

### [^11^C]prucalopride biodistribution

The biodistribution of radioactivity following IV injection of [^11^C]prucalopride was measured in male Wistar rats using a modification of the method described by Airaksinen et al. [18]. Under 2% isoflurane anaesthesia, four groups of four rats received an intravenous injection (tail vein) each of [^11^C]prucalopride (53 ± 2 MBq at the start of the experiment). Rats (four per time point) were killed, under anaesthesia, by cervical dislocation at 5, 15, 30 and 60 min post injection. Whole blood was collected by cardiac puncture, and the heart, lungs, liver, colon, kidneys and brain were excised. The following brain regions were dissected: olfactory bulb, hippocampus, striatum, frontal and posterior cortex (including occipital cortex), thalamic region, medulla oblongata, cerebellum and the rest of the brain. All organs and brain regions were weighed and counted for radioactivity using an automated gamma counter. Values were corrected for decay and the percentage of the total injected radioactivity dose (%ID g^−1^) for every tissue was calculated as percentage of the total injected radioactivity dose divided by the weight of the tissue.

### *In vivo* stability of [^11^C]prucalopride

Wistar rats (two groups of three) received an intravenous injection of [^11^C]prucalopride (100 ± 40 MBq at the start of the experiment) via the tail vein under 2% isoflurane anaesthesia. At 5 and 30 min post injection, whole blood was collected by cardiac puncture and the brain was excised. Whole blood was centrifuged for 5 min at 4,000 rpm, and a 1-mL plasma aliquot was taken and acidified using 10 μL of 5 M HCl and diluted with 2 mL of water. The excised brain was washed in ice-cold saline and subsequently, one hemisphere was homogenized in 12 mL ice-cold saline and centrifuged for 5 min at 4,000 rpm. Plasma and brain extracts were passed over Waters tC18 Plus Sep Pak® cartridge (1 g, pre-conditioned with 10 mL ethanol followed by 10 mL water), washed with 5 mL water and eluted with 2 mL acidic methanol. All fractions, including the Sep Pak® cartridge, were counted for radioactivity using an automatic gamma counter. The methanol fraction was analysed using the semi-preparative HPLC method for the presence of parent [^11^C]prucalopride.

### [^11^C]Prucalopride PET studies

#### PET system

PET measurements were performed using a high-resolution research tomograph (HRRT) (CTI/Siemens, Knoxville, TN, USA) scanner. The HRRT is a dedicated human brain PET scanner, with design features that enable high spatial resolution combined with high sensitivity, making it also suitable for small animal imaging. This scanner has a transaxial field of view of 312 mm and an axial field of view of 250 mm. The spatial resolution ranges from 2.3 to 3.2 mm full width at half maximum (FWHM) in transaxial direction and from 2.5 to 3.4 mm FWHM in axial direction [19].

#### Rat treatment and PET measurements

Anaesthesia was induced and maintained by constant insufflation of 1.5% to 2% isoflurane in pure oxygen into the nose. Rats were placed in a fixation device with tooth bar to secure a fixed and immobile horizontal position of the head during scanning. Body temperature was kept constant at 37°C with a heating pad coupled to a thermostat which was connected to a rectal thermometer. A cannula was inserted into the vena femoralis for radiotracer injection. Transmission measurements of 6 min duration were performed using a 740 MBq 2D fan-collimated ^137^Cs moving point source.

A bolus of 10.6 ± 2.6 MBq of [^11^C]prucalopride was injected IV. A 3D emission acquisition at 45-min duration was started immediately prior to the IV injection. Four rats underwent two consecutive PET scans at 30-min interval. The first PET scan was performed under baseline conditions, the second PET scan was performed following pre-treatment with tariquidar, 15 mg·kg^−1^ IV given as slow bolus 30 min prior to the [^11^C]prucalopride injection. At the end of the [^11^C]prucalopride PET scans, [^18^F]NaF (approximately 15 MBq) was injected and after 30 min, a 30-min emission scan was acquired.

#### Image acquisition and reconstruction

Acquired [^11^C]prucalopride data were stored in 64-bit list mode format and were subsequently histogrammed into 21 time frames of 7 × 10, 1 × 20, 2 × 30, 2 × 60, 2 × 150 and 7 × 300 s duration. The [^18^F]NaF data were stored as a single 30-min frame. All data were normalized and corrected for attenuation, randoms, scatter, decay and dead-time. Data were reconstructed using 3D ordered subsets-weighted least squares (3D-OSWLS) [20] using seven iterations and 16 subsets. The image matrix was 256×256×207 voxels with a voxel size of 1.218 × 1.218 × 1.218 mm^3^.

#### PET data analysis

##### Region of interest analysis

[^18^F]NaF scans of the brain were co-registered with a standard MR template to delineate regions of interest (ROIs) as previously described [21]. The following ROIs were used: striatum (30 mm^3^), hippocampus (27 mm^3^), frontal plus parietal cortical area (49 mm^3^), posterior plus occipital cortical area (49 mm^3^), and cerebellum (86 mm^3^). Peripheral ROIs of the heart, liver, small intestine (jejunum and ileum), colon, and the caecum were manually drawn directly into the dynamic PET images. ROIs were transferred onto the dynamic PET images, and regional time activity curves (TACs in kBq·mL^−1^) were generated [22].

##### Binding potential quantification

The simplified reference tissue model [23], with cerebellum as reference tissue, was used to calculate non-displaceable binding potential (BP_ND_) as outcome measure of specific binding in the analysed brain regions [24].

##### Statistical analysis

Differences in BP_ND_ values between baseline and post tariquidar scans were tested using a general linear mixed model in SPSS Statistics (version 17.0; SPSS Inc., Chicago, IL, USA). All results are expressed as mean ± standard deviation (SD), and values of *p* < 0.05 were considered to be statistically significant.

## Results

### Radiolabelling and automated synthesis of [^11^C]prucalopride

**Figure 1:**
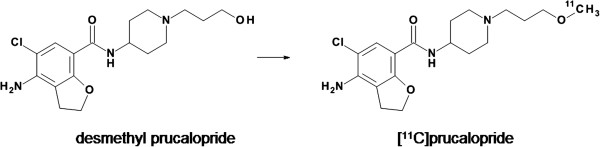
**Chemical structures of desmethyl prucalopride and [**^**11**^**C]prucalopride.** Optimized reaction conditions and reagents for the radiosynthesis are as follows: [^11^C]methyl triflate, CH_3_CN, TBAOH, 5 min and 85°C.

Optimized radiosynthesis of [^11^C]prucalopride (Figure 1) followed by preparative purification on HPLC, isolation by solid phase extraction and formulation, yielded 1.1 to 1.3 GBq of formulated [^11^C]prucalopride. Total preparation time was 45 ± 5 min, yield 21% to 25%, radiochemical purity >99% and SA 52 ± 19 GBq·μmol^−1^. Radiochemical yields for several reaction conditions and parameters investigated are presented in Table 1. These yields are decay corrected and either determined by analysing samples taken from the reaction mixture (analytical yields) or from activities at the start of synthesis and in the formulated product.

**Table 1:** **Overview of parameters investigated to optimize radiosynthesis of [**^**11**^**C]prucalopride**

**Amount desmethyl prucalopride (μmol)**	**Base**	**Amount base (μmol)**	**Temp (°C)**	**Methylation agent**	**Yield (%)**	**Yield**^**a**^	**Number**
3.0	NaOH	25.0	25	[^11^C]CH_3_I	0	Analytical	1
3.0	NaOH	25.0	85	[^11^C]CH_3_I	1	Analytical	1
3.0	NaOH	5.0	25	[^11^C]CH_3_I	6	Analytical	1
3.0	NaOH	5.0	85	[^11^C]CH_3_I	7	Analytical	1
2.2	TBAOH	5.1	25	[^11^C]CH_3_I	11 ± 1	Analytical	2
2.2	TBAOH	5.1	85	[^11^C]CH_3_I	12 ± 1	Analytical	2
3.0	TBAOH	6.9	85	[^11^C]methyl triflate	12 ± 0	Analytical	2
2.2	TBAOH	4.4	85	[^11^C]methyl triflate	16 ± 0	Preperative	2
3.5	TBAOH	5.3	85	[^11^C]methyl triflate	31 ± 2	Preperative	2
2.2	TBAOH	3.5	85	[^11^C]methyl triflate	32 ± 1	Preperative	2
2.7	TBAOH	4.3	85	[^11^C]methyl triflate	34 ± 3	Preperative	14

For every reaction, CH_3_CN was used as solvent; the reaction volume was 350 μL and the reaction time was 5 min. ^a^The presented yields are either determined by analysing samples taken from the crude reaction mixture (analytical yields) or determined from the activity at start of synthesis and the activity in the formulated product (preparative yields).

**Figure 2:**
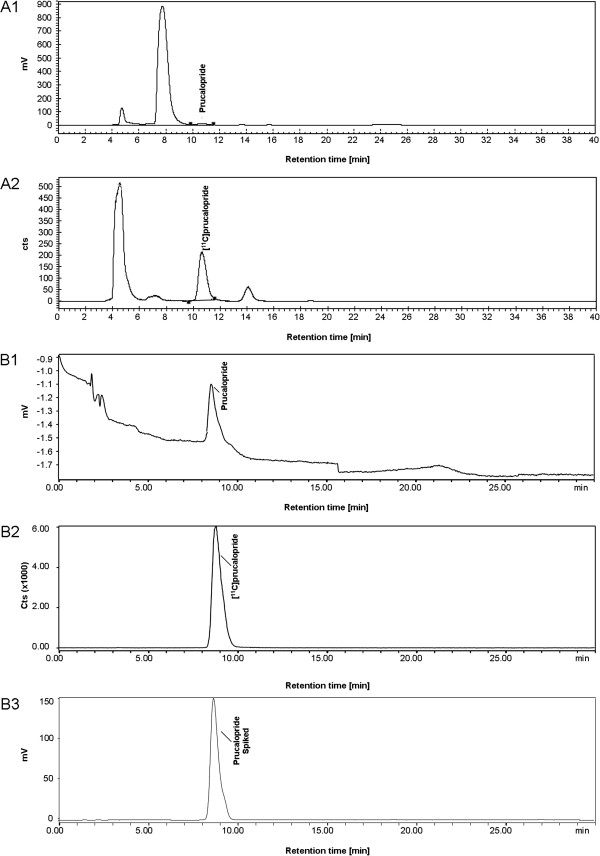
**Representative HPLC chromatograms of the purification of the reaction mixture and formulated [**^**11**^**C]prucalopride.** (**A**) Chromatograms of the semi-preparative HPLC purification of the reaction mixture. (**A1**) UV vs. time; (**A2**) radioactivity vs. time. Retention times were 8 min for the precursor and 11 min for [^11^C]prucalopride. (**B**) Analytical HPLC chromatograms of formulated [^11^C]prucalopride vs. time. (**B1**) UV vs. time; (**B2**) radioactivity vs. time; (**B3**) UV spiked with cold prucalopride. Retention times were 8.5, 8.8 and 8.5 min for UV, radioactivity signal of [^11^C]prucalopride and UV spiked with cold prucalopride, respectively. UV and radioactivity detections were connected in series, giving a time delay of 0.3 min.

The methylation reaction occurred with significant formation of side-product. In addition, there was a significant amount of unreacted [^11^C]methyl triflate (Figure 2). Formation of [^11^C]prucalopride was confirmed by analytical HPLC using co-injection of reference prucalopride. Representative chromatograms of both semi-preparative and analytical HPLC purification are shown in Figure 2.

### LogD_oct pH7.4_ value of [^11^C]prucalopride

The measured LogD_oct,pH7.4_ value of [^11^C]prucalopride was 0.870 ± 0.004 (*n* = 3). The recovery of radioactivity in the sum of 1-octanol and phosphate buffer fractions was 94.1% ± 1.7%.

### [^11^C]prucalopride biodistribution studies

The biodistribution of radioactivity after IV injection of [^11^C]prucalopride in rats was determined *ex vivo* in brain regions, peripheral organs and blood at 5, 15, 30 and 60 min. Results are presented in Table 2. At 5 min post IV injection of [^11^C]prucalopride, low levels of radioactivity in brain were observed, the highest percentage of the total injected radioactivity dose value being 0.13 in the olfactory bulb. Levels of radioactivity in olfactory bulb, striatum and hippocampus were higher than in cortical areas, thalamus, medulla oblongata, cerebellum and the rest of the brain. Over time, radioactivity in brain areas fluctuated over the 60 min, and a decrease of 60% to 70% was observed. In the peripheral tissues, radioactivity levels at 5 min were highest in the kidney followed by the liver and lung; however, the level was lower in the colon and very low in the heart and blood. Radioactivity in the kidney and lung was reduced to 1/3 at 15 min. The disappearance of radioactivity with time was slower for other tissues and, in particular, in the liver.

**Table 2:** Distribution of radioactivity in several brain regions and peripheral organs in rats

**Organ/region**	**Percentage ID/g (mean ± SD, *n *= 4 per time point)**			
	**5 min**	**15 min**	**30 min**	**60 min**
Olfactory bulb	0.13 ± 0.04	0.05 ± 0.03	0.10 ± 0.05	0.04 ± 0.01
Striatum	0.07 ± 0.01	0.05 ± 0.02	0.08 ± 0.05	0.04 ± 0.01
Hippocampus	0.09 ± 0.02	0.05 ± 0.02	0.05 ± 0.01	0.03 ± 0.01
Medulla oblongata	0.06 ± 0.00	0.03 ± 0.01	0.05 ± 0.00	0.03 ± 0.00
Frontal cortex	0.06 ± 0.00	0.03 ± 0.02	0.03 ± 0.00	0.02 ± 0.00
Thalamic region	0.05 ± 0.00	0.03 ± 0.01	0.03 ± 0.01	0.02 ± 0.01
Posterior cortex	0.06 ± 0.01	0.04 ± 0.02	0.03 ± 0.01	0.03 ± 0.01
Rest of the brain	0.06 ± 0.00	0.03 ± 0.02	0.05 ± 0.00	0.03 ± 0.00
Cerebellum	0.08 ± 0.01	0.04 ± 0.02	0.04 ± 0.00	0.02 ±0.00
Liver	1.60 ± 0.58	1.32 ± 0.59	1.53 ± 0.19	0.83 ± 0.05
Kidney	2.57 ± 0.24	1.06 ± 0.49	0.79 ± 0.10	0.43 ± 0.04
Lungs	1.55 ± 0.61	0.51 ± 0.23	0.39 ± 0.13	0.24 ± 0.04
Colon	1.01 ± 0.29	0.54 ± 0.26	0.32 ± 0.07	0.22 ± 0.03
Heart	0.39 ±0.04	0.21 ± 0.10	0.19 ± 0.01	0.10 ± 0.01
Blood	0.16 ± 0.01	0.08 ± 0.04	0.10 ± 0.01	0.06 ± 0.00

Radioactivity is measured *ex vivo* at various time points following IV injection of 53 ± 2 MBq (SA 42 ± 8 GBq·μmol^−1^) of [^11^C]prucalopride. Tissue samples were not flushed with saline prior to the radioactivity measurement. Experiments were performed on two separate days, day 1 at 5 and 15 min and day 2 at 30 and 60 min.

### *In vivo* stability of [^11^C]prucalopride in rats

*In vivo* stability of [^11^C]prucalopride was measured at 5 and 30 min post IV injection of [^11^C]prucalopride, by analysing the presence of the parent compound in plasma and brain methanol extracts using HPLC. The complete procedure was performed with a radioactivity recovery of >90%. Most of the radioactivity was recovered in the water fraction of the Seppak, which would be polar radiolabelled metabolites of [^11^C]prucalopride; however, these products were not identified. The methanol extract of the Seppak column, expected to contain parent [^11^C]prucalopride, contained no parent compound for both plasma and brain extracts taken at 5 and 30 min post injection.

### [^11^C]prucalopride PET studies in rats

**Figure 3:**
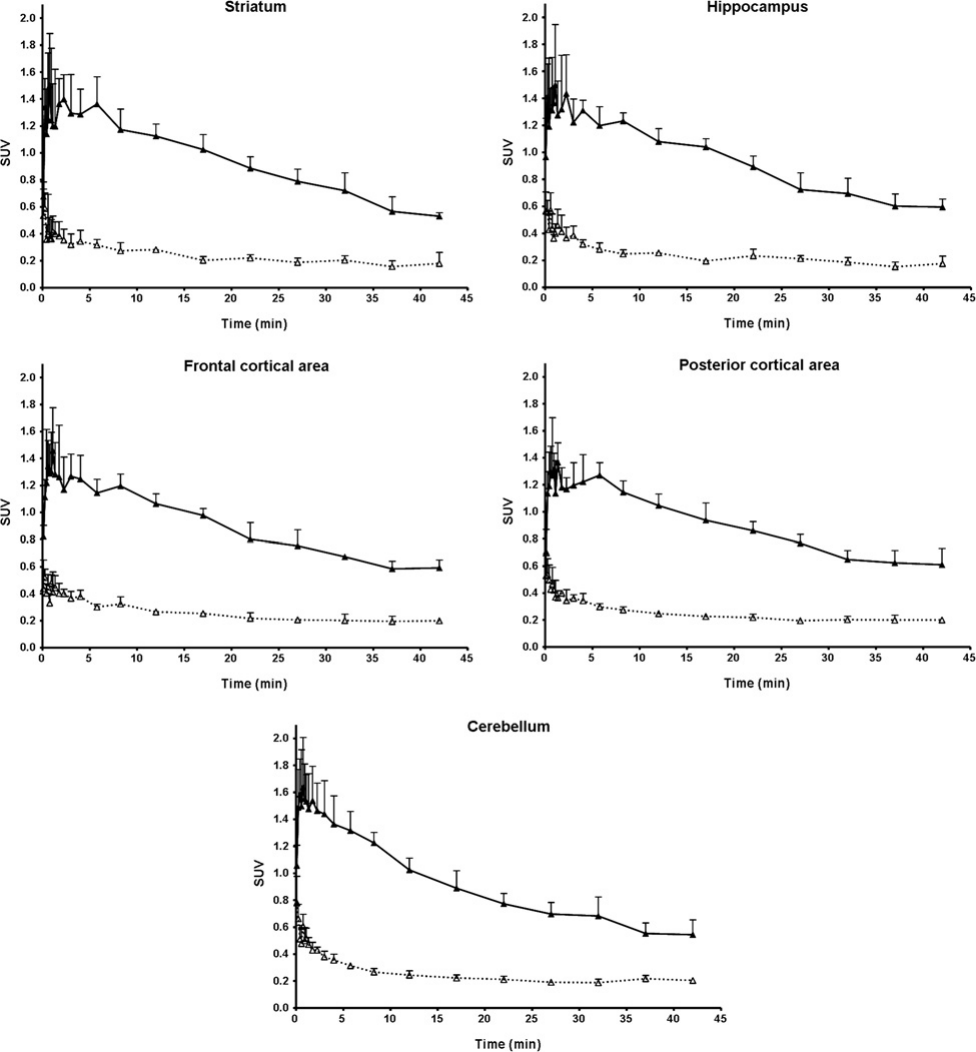
**Time-activity curves in various brain regions, following IV injection of 15±4 MBq [**^**11**^**C]prucalopride.** Time-activity curves in various brain region, measured using PET, following IV injection of 15 ± 4 MBq [^11^C]prucalopride (SA 65 ± 5GBq·μmol^−1^). Open triangles (with dashed line) represent SUVs at baseline, and solid triangles (with solid line) represent SUVs after tariquidar pre-treatment (15 mg·kg^−1^ IV). In both cases *n* = 4.

Following IV injection of [^11^C]prucalopride at baseline conditions, all rats showed low cerebral levels of radioactivity. Radioactivity levels were highest at 30 s, and for analysed brain regions corresponded to a SUV of about 0.6, which was declined to ≤0.3 at 40 min (Figure 3).

**Figure 4:**
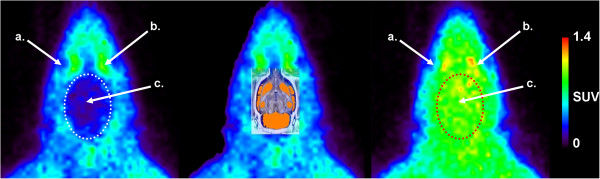
**Summed (0 to 20 min after injection) [**^**11**^**C]prucalopride images.** (left) baseline, (middle) baseline co-registered with MR template and (right) following tariquidar (15 mg·kg^−1^ IV) predosing. Arrows indicate the (**a**) eye, (**b**) Harderian gland and (**c**) brain.

In all animals, tariquidar pre-treatment resulted in higher cerebral concentrations of radioactivity following the IV injection of [^11^C]prucalopride than at baseline. In fact, brain radioactivity concentrations were approximately three-fold higher in all brain regions with a peak SUV of 1.3 ± 0.2, which subsequently declined to SUVs ≤ 1 within 20 min (Figure 3). Representative PET images are shown in Figure 4.

The BP_ND_ values for the striatum, hippocampus, frontal cortex, posterior cortex and medulla oblongata, using cerebellum as reference tissue, are shown in Table 3. At baseline, BP_ND_ values were essentially zero. Following tariquidar pre-treatment, however, positive BP_ND_ values were obtained for the striatum and hippocampus.

**Table 3:** **BP**_**ND **_**for [**^**11**^**C]prucalopride in rat brain regions versus cerebellum**

**Brain region**	**Baseline**	**Tariquidar pre-treatment**
Striatum	0.00 ± 0.08	0.07 ± 0.09**
Hippocampus	−0.05 ± 0.05	0.07 ± 0.05*
Frontal cortex	0.04 ± 0.07	0.03 ± 0.04
Posterior cortex	0.00 ± 0.05	0.03 ± 0.04

BP_ND_ for [^11^C]prucalopride in rat brain regions, at baseline and following pre-treatment with tariquidar (*n* = 4 for both conditions)**.** Significant difference compared with baseline: *p < 0.05, **p < 0.01.

**Figure 5:**
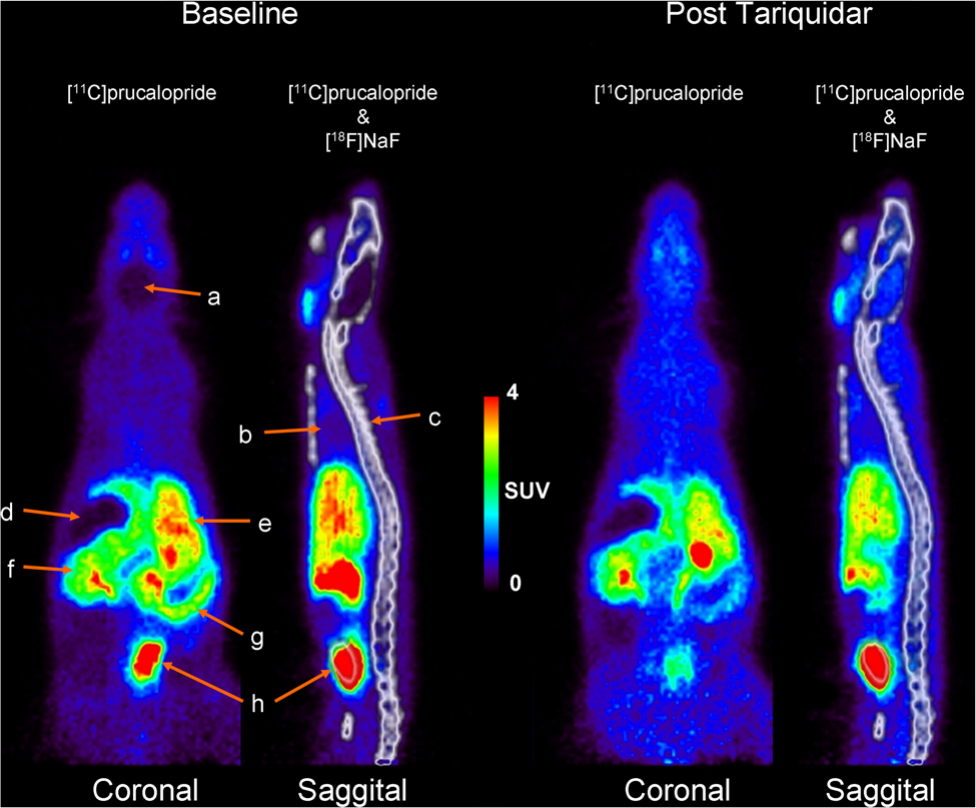
**Summed (0 to 45 min after injection) whole body PET images following IV injection of [**^**11**^**C]prucalopride in rat.** (left) Baseline and after (right) pre-treatment with tariquidar (15 mg·kg^−1^ IV). Arrows indicate the (**a**) brain region, (**b**) lungs, (**c**) spine, (**d**) stomach, (**e**) liver, (**f**) jejunum, (**g**) caecum, and (**h**) bladder.

**Figure 6:**
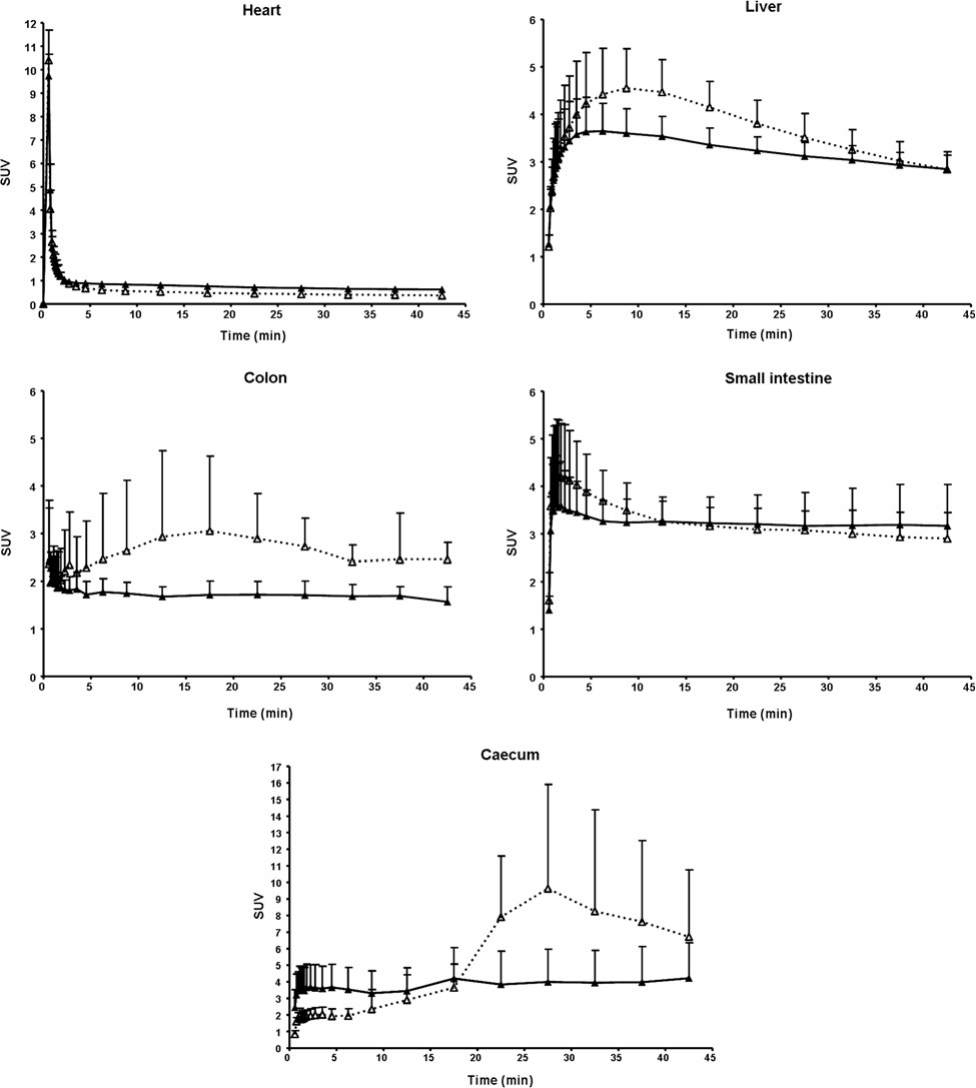
**Time activity curves in selected peripheral tissues following IV injection of 15±4 MBq [**^**11**^**C]prucalopride.** Time activity curves in selected peripheral tissues following IV injection of 15 ± 4 MBq [^11^C]prucalopride (SA 65 ± 5 GBq·μmol^−1^). Open triangles (with dashed line) represent SUVs at baseline; solid triangles (with solid line) represent SUVs after tariquidar pre-treatment (15 mg·kg^−1^ IV). Data represent mean ± SD (*n* = 4).

The representative whole body PET images following IV injection of [^11^C]prucalopride at baseline and after pre-treatment with tariquidar (15 mg·kg^−1^ IV) are shown in Figure 5. High levels of radioactivity over time (summed image 0 to 45 min) were seen in the bladder, liver, jejunum, colon, and caecum (the beginning of the colon). Time activity curves based on SUV values in selected tissues are shown in Figure 6. The hearth showed high SUV values (maximal SUVs of 10.4 ± 1.3) only within the first minute after IV injection of [^11^C]prucalopride, whereafter SUV values were <1. Tariquidar pre-treatment did not affect the time activity curve of SUV values in the heart. In liver a maximal SUV of 4.6 ± 0.8 was observed at 8.75 min post injection which slowly declined over time. Pre-treatment with tariquidar afforded lower maximal SUVs of 3.6 ± 0.4 which declined to 2.8 ± 0.3 at the end of the scan. Radioactivity levels in the small intestine were initially higher for baseline compared to post tariquidar treatment, after 12.5 min SUVs were approximately 3.3 under both conditions and remained constant over time. For the colon, levels of radioactivity under baseline were maximal at 17.5 min with SUVs of 3.1 ± 1.6 which declined to SUVs of 2.5 ± 0.4 at the end of the scan. Post tariquidar colon SUVs were approximately 1.8 over time. The ROI placed on the caecum showed that SUVs, at baseline conditions, increased to maximal SUVs of 9.6 ± 6.2 at 27.5 min post injection. Post tariquidar measurements afforded SUVs of approximately 3.5, which remained constant over time.

## Discussion

### Radiolabelling and automated synthesis

Radiolabelling of [^11^C]prucalopride was performed using both [^11^C]CH_3_I and [^11^C]methyl triflate. Using NaOH as a base, [^11^C]CH_3_ incorporation was low and labelled side products (80% to 90%) were formed primarily. Using TBAOH as the base resulted in less side products and incorporation of [^11^C]CH_3_ of 7% to 11%, possibly due to the reduced deprotonation of the secondary amine group of the precursor. Using [^11^C]methyl triflate and still lower amounts of TBAOH further increased [^11^C]CH_3_ incorporation. Optimal conditions were achieved with 1.5 to 1.6 molar equivalents of base, resulting in an incorporation yield of 30% to 36% of [^11^C]CH_3_. Using these optimal conditions, automated radiosynthesis resulted in a formulated product with a total yield of 21% ± 4% (decay corrected) at the end of an overall preparation time of 45 ± 5 min, with good specific activity and high radiochemical purity.

### LogD_oct, pH7.4_ value of [^11^C]prucalopride

LogD was used as parameter for lipophilicity rather than LogP, as LogP relates to lipophilicity for the charge-neutral form of the radioligand only, whilst LogD takes into account the sum of unionized and ionized forms of prucalopride that contains a primary, secondary and tertiary amine functions at a physiological pH of 7.4 [25]. The measured LogD_oct,pH7.4_ value of 0.87, corresponding to a distribution ratio of 7.76 between 1-octanol and phosphate buffer of pH 7.4, points to a relatively low lipophilic nature of prucalopride. Pike reported in 2009 that PET ligands with moderate lipophilicity indicated by logD_oct,pH7.4_ values in the range of 2.0 to 3.5 showed optimal passive brain entrance. Exceptionally, some useful radiotracers with lower or higher logD_oct,pH7.4_ values also entered the brain, but mostly for unclear reasons [26]. The relatively low lipophilicity of prucalopride may hamper its passive diffusion into the brain.

### [^11^C]prucalopride stability *in vivo*

In this *in vivo* stability study in rats, the parent [^11^C]prucalopride was not detectable in the blood and brain extracts, at 5 or 30 min following IV injection; whereas different radiolabelled products were detected at both time points. This confirms the findings of previous studies, showing that in male rats, prucalopride is extensively metabolised, reportedly through hydroxylation and/or *O*-demethylation. Such extensive metabolism was not seen in other species (Shire-Movetis, unpublished results; partly reported in [27]). In this study, hydroxylation of [^11^C]prucalopride would yield a radiolabelled metabolite which is highly hydrophilic (cLogP 0.007) and is thus expected not to pass the blood-brain barrier readily. *O*-demethylation of [^11^C]prucalopride by CYP1A2 and other isoenzymes in the liver [28-30] would yield unlabelled hydroxylated prucalopride and [^11^C]CH_3_OH and possibly [^11^C]CH_2_O. Formation of the latter was demonstrated in different enzymatic de-methylation reactions and it was found that CH_2_O formed in tissue, readily and spontaneously forms condensation products e.g., with aryle-thylamines, such as catecholamines and indoleamines [31,32]. [^11^C]CH_3_OH and condensation products of [^11^C]CH_2_O may have contributed to nonspecific levels of radioactivity in the brain and peripheral tissues.

### Is [^11^C]prucalopride a potential PET ligand?

Rats, commonly used and readily available species for initial evaluation of potential PET ligands, were also used for the present *ex vivo* biodistribution and *in vivo* PET studies. Unfortunately, evaluation of [^11^C]prucalopride as a potential PET ligand in the rat was hampered by rapid metabolism, in which the male rat is much faster than in other species [27]. Nevertheless, some interesting findings were made. Information was obtained that can explain the low radioactivity levels in the brain of [^11^C]prucalopride in this study. In rats, the brain levels of radioactivity following IV injection of [^11^C]prucalopride was extremely low and under baseline conditions probably represented minute to no parent [^11^C]prucalopride, due to the fast metabolism. Surprisingly, the following pre-treatment with tariquidar levels of radioactivity in all brain areas was increased three-fold within seconds after IV injection of [^11^C]prucalopride (Figure 3). Tariquidar is an inhibitor of the P-glycoprotein ABC transporter. This transporter is located in capillary endothelial cells of the blood-brain barrier, where its function is to translocate xenobiotics out of the brain and in the intestinal epithelium, where it translocates toxic metabolites and xenobiotics from the cells and blood into the intestinal lumen [33,34]. Tariquidar is also an inhibitor of CYP1A2, an enzyme involved in the metabolism of prucalopride. Therefore, increased levels of radioactivity in the brain following tariquidar pre-treatment could be a result of slower metabolism and/or higher blood concentrations of unchanged [^11^C]prucalopride. On the other hand, it may also indicate that prucalopride is a P-glycoprotein substrate and that its removal from the brain is reduced by inhibition of the pump. The extremely fast appearance of the effect could suggest that the radioactivity that appears in the brain within seconds after injection represents for the major part parent [^11^C]prucalopride. Hence, low levels of radioactivity in rat brain under baseline conditions could be due to (1) rapid metabolism, in particular in male rats, (2) limited passive diffusion owing to its low lipophilicity, and (3) the possibility of being a P-glycoprotein substrate. In a pilot PET study in one pig, the parent compound was detected in the blood with limited to no uptake in brain (unpublished observation), supporting the low brain uptake of [^11^C]prucalopride .

Radioactivity levels in peripheral tissues following IV injections of [^11^C]prucalopride were substantially higher than in the brain, as shown in both the *ex vivo* biodistribution and in the *in vivo* PET study. PET images in the gut deserve particular attention in view of the therapeutic application of prucalopride in gastrointestinal motility disorders. In this rat PET study, the caecum could clearly be delineated, although no analysis was done on the identity of the radioactivity in the gut. SUV values in the caecum and colon as a whole were reduced after tariquidar pre-treatment as compared to the baseline. This is in line with the role of the P-glycoprotein pump in the gut [35]. If prucalopride is a P-glycoprotein substrate, its transport from blood into the intestinal lumen should indeed be reduced by inhibiting P-glycoprotein.

5-HT_4_-R has been localized in the colonic mucosa and circular muscles [36]. As prucalopride is used to treat constipation, the ability to investigate the active state of 5-HT_4_-R in the colon and the intestine in general, *in vivo*, would be highly interesting. It could provide information on the active 5-HT_4_-R, for example, in cases of reduced gastric motility and it would allow monitoring possible 5-HT_4_-R desensitisation during treatment with 5-HT_4_-R agonists. Further evaluation of [^11^C]prucalopride as a potential agonist PET ligand for 5-HT_4_-R in humans, where prucalopride is slowly metabolised [2], seems worthwhile. In human studies, the labelling of peripheral 5-HT_4_-R could be fully explored and the uptake into the brain could be further checked.

## Conclusions

[^11^C]prucalopride was successfully synthesized. However, because of its extremely fast metabolism, the male rat appeared not an appropriate species to assess the value of [^11^C]prucalopride as PET ligand. Because of low lipophilicity and the possibility of it being a P-glycoprotein substrate, [^11^C]prucalopride may not be suitable for *in vivo* imaging of central 5-HT_4_-R. However, further investigation of [^11^C]prucalopride for imaging the active state of 5-HT_4_-R, in particular, in human, is worthwhile in view of therapeutic applications of 5-HT_4_ agonists for the treatment of gastrointestinal motility disorders.

## Competing interest

The HJCB, ADW, MCH, AAL and JEL declare that they have no competing interests. The study was financed by a grant from the A.J. Coops Foundation. No financial support was obtained from Shire-Movetis. JHDM and JAJS are employees of Shire-Movetis, the company that markets prucalopride (Resolor®) in Europe.

## Authors’ contributions

HJCB performed the Carbon-11 tracer synthesis and animal studies. Data analysis and modelling was performed by HJCB and MCH. Statistical analysis of the outcome measures was performed by HJCB. HJCB, ADW, MCH, JHDM, JAJS and JEL participated in the study design, coordination and discussion of findings. HJCB drafted the manuscript with participation of MCH, ADW, JHDM, JAJS and JEL. ADW, MCH, JEL, JHDM, JAJS and AAL proofread the manuscript. All authors read and approved the final manuscript.
